# Persistent pharmacokinetic challenges to pediatric drug development

**DOI:** 10.3389/fgene.2014.00281

**Published:** 2014-08-27

**Authors:** Daniel P. Sage, Christopher Kulczar, Wyatt Roth, Wanqing Liu, Gregory T. Knipp

**Affiliations:** ^1^Department of Industrial and Physical Pharmacy, College of Pharmacy, Purdue UniversityWest Lafayette, IN, USA; ^2^Department of Medicinal Chemistry and Molecular Pharmacology, College of Pharmacy, Purdue UniversityWest Lafayette, IN, USA

**Keywords:** pediatric, ontogeny, pharmacokinetic, ADME, PBPK

## Abstract

The development of new therapeutic agents for the mitigation of pediatric disorders is largely hindered by the inability for investigators to assess pediatric pharmacokinetics (PK) in healthy patients due to substantial safety concerns. Pediatric patients are a clinical moving target for drug delivery due to changes in absorption, distribution, metabolism and excretion (ADME) and the potential for PK related toxicological (T) events to occur throughout development. These changes in ADMET can have profound effects on drug delivery, and may lead to toxic or sub-therapeutic outcomes. Ethical, economical, logistical, and technical barriers have resulted in insufficient investigation of these changes by industrial, regulatory, and academic bodies, leading to the classification of pediatric patients as therapeutic orphans. In response to these concerns, regulatory agencies have incentivized investigation into these ontogenic changes and their effects on drug delivery in pediatric populations. The intent of this review is to briefly present a synopsis of the development changes that occur in pediatric patients, discuss the effects of these changes on ADME and drug delivery strategies, highlight the hurdles that are still being faced, and present some opportunities to overcome these challenges.

## INTRODUCTION

Dr. Harry Shirkey of the Children’s Hospital in Alabama originally described pediatric populations as “therapeutic orphans” in a 1962 manuscript because of children’s neglected status during drug development (Shirkey, 1968). Approximately 50 years later, the aversion to developing new therapeutic agents for children still stands. This aversion is attributable to a variety of factors, including ethical concerns associated with testing drugs in children, the lack of financial return on investment due to smaller patient populations, biological challenges resultant of physiological maturation/development, lack of centers with trained clinicians to conduct pediatric clinical trials, and formulation challenges to improve patient compliance (Milne and Bruss, 2008). In response to these challenges, the United States FDA has issued legislation such as the Best Pharmaceuticals for Children Act (BPCA) and the Pediatric Research Equity Act (PREA) to provide incentives for testing and developing drugs for children. Similar incentives and initiatives have been advanced by regulatory agencies throughout the world. While these incentives have significantly improved pediatric drug labeling and increased the number of clinical trials being performed in children, a large unmet need still exists for the development of child-friendly dosage forms (Hoppu et al., 2012). Toward this point, the World Health Organization estimated that more than 7.6 million deaths in children under the age of 5 occurred worldwide in 2010, with greater than 99% of these deaths occurring in low to middle income countries. While some of these deaths are the result of prenatal complications, the yearly number of preventable childhood deaths, e.g., symptoms of communicable diseases, still lies in the millions. Clearly, the therapeutic intervention of pediatric disorders remains a critical area for continued research.

The pediatric population, especially in the neonatal to infant stages of development, represent a dynamic group in respect to physiology. Through the recognition brought on by the legislation described above there has been an increase in activity to seek answers to the challenges faced in determining how to safely and effectively deliver medicines to children (Gazarian, 2009). From this activity, many questions have begun to be answered, but many still have yet to be addressed. Throughout this review, we will present challenges associated with and opportunites to develop age appropriate peroral drug dosage forms for children, particularly in the neonatal, infant, and toddler stages of development. Hence, we will dissect and highlight some of the significant challenges that must be overcome in order for investigators to make significant inroads in the field.

Traditionally, pediatric doses are developed through allometric scaling of adult doses using metrics such as ratios of body surface area or body weight (Bartelink et al., 2006; Gazarian, 2009; Tucker, 2009; Ansel, 2010; De Cock et al., 2011). For example, initial doses are often calculated from adult clearance values, which factors in endogenous increases in liver volume by taking body weight to the 3/4 power (Bartelink et al., 2006; Gazarian, 2009; Tucker, 2009; Ansel, 2010). The principal limitation to this approach is that this method is only useful in later stages of child development, since it only accounts for changes in organ size and assumes that the pharmacokinetic and pharmacodynamic properties are the same (Ansel, 2010). This is an invalid assumption in earlier developmental stages since the morphology and ontogeny of critical drug metabolizing and transporting isoforms and their regulatory systems change in organs from the neonatal to infant stage of childhood (De Wildt et al., 1999; Johnson, 2003; Kearns et al., 2003; Björkman, 2005; Bartelink et al., 2006; Gazarian, 2009; Kramer, 2009; Tucker, 2009; De Cock et al., 2011). Therefore, developmental changes are a considerable concern and may have significant effects that render ratio calculations useless for younger children, leading to the phrase that children are not little adults (De Wildt et al., 1999; Milne and Bruss, 2008).

It is currently recognized that the research into the design of pediatric doses must be predicated on the acquisition of data using a combination of bottom up and top down methods (De Wildt et al., 1999; Kearns et al., 2003; Björkman, 2005; De Cock et al., 2011). Bottom up methods use diverse and discrete pieces of prior physiological and genetic information together with *in vitro* data to rationally try and anticipate *in vivo* changes in pediatric dose safety and efficacy. The use of physiological based pharmacokinetic (PBPK) modeling approaches can aid in the extrapolation of pediatric doses made in the bottom up stages (De Wildt et al., 1999; Kearns et al., 2003; Björkman, 2005; Ansel, 2010; De Cock et al., 2011). The bottom up approach can be used to select a dose or a range of doses, which are then confirmed to be safe and efficacious by top down methods. Top down methods rely on the administration of doses and use of the plasma concentration versus time data from the population of interest to assess if co-variates such as age result in predictable changes. It is important to note that many of the clinical studies are first conducted in adults. Furthermore, this approach is utilized when either a significant history of adult use of the medication is present or when there is a significant risk from the disease that outweighs the concerns arising for potential drug-related adverse effects.

## PHYSIOLOGICAL CONSIDERATIONS REGARDING PEDIATRIC PHARMACOKINETICS

The PK of a drug is predicated on the absorption, distribution, metabolism, and excretion (ADME) of an individual drug upon administration. The goal of any dosing regimen is to provide consistent and predictable systemic drug exposure drug in the patient resulting in a safe and efficacious response. Ontogenic morphological and drug metabolizing and transporter expression changes have a profound effect on ADME, particularly in the first 18 months of life (Kramer, 2009). In fact, these changes influence all parts of ADME. For instance, absorption through the GI tract may be affected by changes in morphology of the GI epithelium, increased body fat percent in pediatric patients may influence distribution of lipophilic drugs, changes in CYP450 expression can alter drug metabolism rates, and development of kidney function in pediatric patients can impact excretion in the urine. Because of these rapid changes in growth and development, it is extremely difficult to give general dosing requirements particularly for prenatal, infant, and toddler populations (De Wildt et al., 1999; Johnson, 2003; Kearns et al., 2003; Björkman, 2005; Bartelink et al., 2006; Kearns, 2009; Kramer, 2009; Tucker, 2009; De Cock et al., 2011). We will highlight some representative examples of physiological changes that influence ADME during different stages of development to demonstrate how these differences impact decisions on the drug dose. It must be emphasized that these generalizations are based on assumptions that may not apply to each specific drug, the specific disease state being treated, or other potential factors that have not yet been fully realized. Specific dosage regimens from this model simply act as a guide when no data exist and/or for doing clinical testing that will be discussed in the next section.

### ABSORPTION

A drug administered extravascularly must overcome a variety of chemical, physical, mechanical, and biological barriers to be absorbed. The extent of drug absorption from a dosage form is referred to as the bioavailability. In general, absorption across the gastrointestinal (GI) epithelium occurs via either a single or a combination of several permeation routes including the paracellular route (hydrophilic, diffusion between cellular junctions), passive transcellular (lipophilic, across the lipid bilayers of the apical and basolateral membranes), by active transporters (influx or eﬄux), metabolism, and in some cases endocytosis. We will not elaborate upon these routes for the sake of brevity.

In the neonatal, infant, and toddler age groups, peroral formulations are often solution based such as syrups or suspensions. Solid dosages are often avoided based on the difficulty that these age groups have in swallowing, yet they may offer a considerable safety advantage when considering differences in the rates of drug degradation in solutions vs. solids. The development of sprinkles, oral dissolving films and oral disintegrating tablets do offer promise to overcome the stability challenges.

Peroral drug absorption is primarily affected by developmental changes in the GI tract. Perhaps the most commonly discussed difference in the neonatal period is the elevation of intragastric pH, which can affect the ionization, stability and absorption of different drugs (Kearns et al., 2003; Bartelink et al., 2006; Kramer, 2009). Gastric pH values are in the range of 6–8 at birth, and drop within hours to 2–3. However, after the first 24 h of life, the gastric pH begins to elevate to more alkaline levels due to the immature parietal cells which are responsible for secreting gastric acid (Kearns et al., 2003). Other GI differences that change with development include: variable effects on gastric emptying and intestinal transit time, which are often prolonged through childhood due to reduced motility; immature biliary function, leading to reduced ability to solubilize lipophilic drugs including vitamins; reduction in intestinal metabolizing enzymes, which may lead to a higher fraction absorbed for some drugs; reduced splanchnic blood flow, which could lead to decreased permeation from the cells of the GI tract to the bloodstream; and a reduction in the intestinal microflora (Johnson, 2003; Kearns et al., 2003; Bartelink et al., 2006; Kearns, 2009; Kramer, 2009). The expression level of drug transporters has not been fully explored, but is a topic of considerable interest in the field (Kearns et al., 2003). There are two generalizations that can be made concerning development stage dependent changes in transporter expression and function: (1) diet plays a critical role in controlling the relative transport activities over time; (2) there is a general decrease in the transporter activity as a function of intestinal surface area due to the large increase in surface area that occurs throughout development (Buddington, 1992).

These physiological changes are representative of a variety of effects that may alter the oral absorption of a drug from a formulation. Moreover, oral administration of drugs to neonates, especially in the first 2 weeks, is generally changed to account for a reduced bioavailability unless there is a significant first-pass effect or if the drug is acid-labile (Bartelink et al., 2006). It is also well known that gastric retention and increased GI motility alter the absorption of enteric coated formulations administered in infants up to about 3–4 months of age. Finally, rectal administration, while common for compounded drugs, is cautioned due to high amplitude pulsatile contractions in the rectum that can increase the chance of dosage form expulsion in early stages of development (Kearns et al., 2003; Bartelink et al., 2006).

### DISTRIBUTION

In general, development differences in the rates of maturation of organs, blood perfusion, percentage of extravascular water, body fat percentage, differential permeation rates into tissues and disease states predominantly influence drug distribution in pediatric patients. Organ maturation is particularly important in neonates and infants, as there are continuous changes until the organs begin to reach maturation. Permeation will vary as barriers to perfusion across the pediatric endothelium are also continuously changing until the cells reach a level of stability (Hoppu et al., 2012).

Drug distribution may be affected by changes in body composition, such as total body water and adipose tissue that may not necessarily be related to changes in the total body weight. As the newborn matures from a neonate, two major changes occur in that the extracellular water decreases and body fat increases (De Wildt et al., 1999). For example, the extracellular water drops from 40% of total body weight at birth to about 20% within in 4 months, thus altering the volume of distribution (V_D_) in hydrophilic, low plasma protein binding drugs. Alternatively, the increases in the percentage of body fat can cause highly lipophilic drugs to have an increased V_D_ during this time. The levels of these body fluids continue to fluctuate throughout the first 3 years of life, before they level off (De Wildt et al., 1999).

Plasma protein binding and tissue binding changes also arise from changes in body composition with growth and development that may influence the V_D_ and clearance (De Wildt et al., 1999). In general, there is a decrease in protein binding due to a decreased amount of plasma proteins, like α1-acid glycoprotein, and a fetal form of albumin that has a decreased affinity for weak acids. Beyond that, an increase in circulating bilirubin leads to a reduction in binding sites. Depending on the drug, this can alter the fraction unbound and affect the safety and efficacy of medicament. For example, in a study to determine the PK of midazolam, the fraction unbound was higher in infants at 0.031 compared to adult values of 0.024 (Björkman, 2005). Traditionally, we focus on the passive transcellular (lipophilic) and paracellular (hydrophilic) routes of permeation. Interestingly, research has indicated a progressive increase in the ratios of the brain to plasma phenobarbital ratios from 28 to 39 weeks. The authors suggested the results indicate the increase in brain uptake is potentially due to changes in blood flow (perfusion) and pore density (paracellular distribution) across the blood-brain barrier (Painter et al., 1981). There is an excellent database maintained by Workman et al. (2013) that provides more detailed information regarding developmental changes in the human brain and potential correlations with other species for scaling purposes^1^.

### METABOLISM

Amongst the greatest changes that occur and influence pediatric PK in the early stages of development is their ability to metabolize drug molecules (De Wildt et al., 1999; Johnson, 2003; Kearns et al., 2003; Kearns, 2009). Drug metabolism usually occurs in the liver, but may also occur in the blood, GI wall, kidney, lung, and skin (Kearns, 2009). We will focus on hepatic metabolism, but it should be noted that there are also significant changes in systemic metabolism that occur during development. In general, metabolism refers to the biotransformation of drug entities by enzymes, although transporters are sometimes included under metabolism. There are several excellent, more comprehensive reviews of drug metabolizing enzyme ontogeny in the literature.

As a brief review, the primary reason for metabolism is to make lipophilic xenobiotics more polar for excretion from the body as opposed to accumulation in fatty tissues (Coleman, 2006). Phase I metabolism generally refers to oxidative enzymes, usually in the Cytochrome P450 (CYP) family, that lead to oxidative reactions. However, non-CYP mediated oxidative metabolism, e.g., hydrolyses, can also be catalyzed by Phase I enzymes (Coleman, 2006). Phase II metabolism is generally referred to as metabolism by addition, where a functional group(s) is often targeted for conjugation of hydrophilic molecules. Phase III metabolism is excretion of the metabolite by eﬄux transporters. Phase I, II, and/or III metabolism can occur in concert to detoxify cells by removing lipophilic xenobiotics and dramatically influence ADME (Coleman, 2006). Based on *in vitro* and *in vivo* studies, three potential ontogenic changes in the expression of these enzymes are observed during development: (1) the enzyme might exhibit high expression during or shortly after birth, followed by a significant reduction in expression levels during the first years of life; (2) the enzyme might have a relatively constant expression levels maintained throughout gestation with minimal changes occurring postnatally; or (3) the enzyme may not be expressed, or expressed at low levels, in the fetus with substantial increases in enzyme level during the first years of life (Kearns, 2009).

The Cytochrome P450 isoforms are the most studied enzymes in humans, with the CYP1, CYP2, and CYP3 subfamilies commonly associated with drug metabolism (De Wildt et al., 1999). The CYP3A family is the most abundant subfamily and displays interesting patterns of expression from birth into adulthood. For example, studies have illustrated that CYP3A4 mRNA, the most common CYP3A isoform found in adults, is virtually undetectable in infants (De Wildt et al., 1999). Whereas, the CYP3A7 isoform, which has relatively low to undetectable levels in adults, constitutes approximately 32% of the total CYP content in the early human fetal liver (De Wildt et al., 1999; Johnson, 2003; Kearns et al., 2003; Kearns, 2009). Interestingly, the expression levels of CYP3A7 quickly decline within the first week of life and are replaced by a gradual increase in the level of CYP3A4 expression. Subsequently, this may have little effect on hepatic clearance for many drugs because the actual level of the entire subfamily of CYP3A remains fairly constant throughout this period and CYP3A4 is 90% homologous to CYP3A7 (De Wildt et al., 1999). However, further clarification is needed. Nonetheless, the results of a study examining the PK of midazolam demonstrated that the CYP3A4 and 3A7 ontogeny affected clearance (Payne et al., 1989). In this study, it was determined that CYP3A7 had a drastically reduced affinity for this drug compared to CYP3A4, which resulted in weight adjusted clearance that followed the ontogenic expression of CYP3A4. Other CYP enzyme changes that occur during development include 1A2, 2B6, 2C8, 2C9, 2C19, 2D6, and 2E1 (Johnson, 2003; Kearns et al., 2003; Kearns, 2009). Each of these CYP isoforms have similar expression patterns as observed with CYP3A4, where they are expressed at low levels at birth and gradually increase to a steady state within the first year of life. A table of the ontogeny of common Phase I enzymes is shown in **Table 1**.

**Table 1:** Phase I drug metabolizing enzymes

Gene	Category	Age upon reaching adult expression
CYP1A1	I	Birth
CYP1A2	III	1M-1Y
CYP2A	III	1M-1Y
CYP2C	III	Birth
CYP2D6	III	Birth
CYP2E1	II	–
CYP2J	II	–
CYP3A4	III	Birth
CYP3A5	III	Birth
CYP3A7	I	1M-1Y
FMO1	I	Birth
ADH1	I	1M-1Y
ADH2	II	Prenatal
ADH3	II	Prenatal

*Ontogenic changes in phase II drug metabolizing enzymes. Categories: I, higher expression before or just after birth with potential decreasing expression throughout development to adulthood.; II, Consistent expression throughout development and potentially throughout adulthood; III, lower expression during beginning of development with potential increasing throughout development to adulthood. Ontogenic Appearance/Disappearance: prenatal, ontogenic changes occur before birth; Birth, ontogenic changes occur at or shortly after birth; 1M-1Y, ontogenic changes occur between first month and year postnatally; –, no change is seen throughout development. Adapted from reference (Hines and McCarver, 2002)*

For the phase II enzymes, there is usually a low level of expression at birth, followed by a gradual increase throughout infancy. For example, several uridine 5′-diphospho-glucuronosyltransferase (UGT) isoforms including UGT1A1, 1A4 and 2B7 have very low hepatic expression at birth (Zaya et al., 2006; Miyagi and Collier, 2007, 2011; Miyagi et al., 2012). UGT1A6 expression and function at birth is believed to be nearly 50% of the adult levels and activity (Miyagi et al., 2012). Interestingly, the expression of UGT1A9 appears to be absent at birth, but the changes in protein expression seemed to correlate with changes in the metabolism of 4-methylumbelliferone specifically inhibited by niflumic acid in children (Miyagi et al., 2012). The correlation did not hold true for adults, where activities did not correlate with protein exposure and environmental factors were proposed to be confounding. Evidence of this can also be observed with morphine through research demonstrating that glucuronidation is detected in infants as young as 24 weeks of age and then quadruples between 27 and 40 weeks, thus requiring a substantial increase in dose to maintain the same level of effectiveness (Kearns et al., 2003). A table of the ontogeny of common Phase I enzymes is shown in **Table 2**.

**Table 2:** Phase II drug metabolizing enzymes.

Gene	Category	Age upon reaching adult expression
GSTA1	II	–
GSTA2	II	–
GSTM	II	–
GSTP1	I	1M-1Y
NAT2	II	–
UGT1A1	III	Birth
UGT1A3	II	–
UGT1A6	III	Birth
UGTB7	II	–
UGTB17	II	–
EPHX1	II	–
EPHX2	II	–
SULT1A1	II	–
SULT1A3	II	–
SULT2A1	III	Prenatal

*Ontogenic changes in phase II drug metabolizing enzymes. Categories: I, higher expression before or just after birth with potential decreasing expression throughout development to adulthood.; II, Consistent expression throughout development and potentially throughout adulthood; III, Lower expression during beginning of development with potential increasing throughout development to adulthood. Ontogenic Appearance/Disappearance: prenatal, ontogenic changes occur before birth; birth, ontogenic changes occur at or shortly after birth; 1M-1Y, ontogenic changes occur between first month and year postnatally; –, no change is seen throughout development. Adapted from reference (McCarver and Hines, 2002).*

Since both phase I and phase II enzymes may be immature, scaling a dose from adults cannot be done during the first 2 months for drugs that have extensive hepatic metabolism (Bartelink et al., 2006; Kearns, 2009). Due to the changes in enzymes, if a drug is metabolized significantly, there needs to be individual drug determinations for how to dose. During the early stages of development, the best approach is to start with a low dose and conduct therapeutic drug monitoring to determine clearance (Bartelink et al., 2006). From two to 6 months, hepatic metabolism starts to increase and conservative doses based on body weight adjustments can begin to be used. Beyond that, doses can be adjusted based on factoring in the expression level of the specific enzyme involved with its metabolism. (Bartelink et al., 2006). It must be cautioned that efficacy monitoring may be more difficult during the early stages of development, and thus a reasonable exposure level to achieve a safe and effective response needs to be established. This can be extrapolated from adult studies, and carries more risk if it is derived from animal models.

### EXCRETION

Drug excretion by the kidney is primarily controlled by glomerular filtration, tubular secretion, and tubular reabsorption. These processes are influenced by nephrogenesis in the infant and the different routes mature at different rates. In the newborn, both glomerular filtration and active secretion take about 6 months to reach full function (Kramer, 2009). In that time, the glomerular filtration rate (GFR) goes from about 13 mL/min/1.73 m (Milne and Bruss, 2008) to 100 mL/min/1.73 m (Milne and Bruss, 2008). Then afterward, children continue develop to become extremely rapid clearers with a GFR close to 130 mL/min/1.73 m (Milne and Bruss, 2008) by age 3. (Kearns et al., 2003). Passive reabsorption of drugs along the proximal tubular walls does not seem to be affected by age. These variables result in the need for individualization of age appropriate doses in drugs that have extensive renal elimination. Therefore, flexible dosing formulations and/or therapeutic drug monitoring are required to maintain safe and efficacious exposure levels. For example, gentamicin is eliminated primarily by glomerular filtration necessitating a decrease in dosing intervals in infants. Inadequate knowledge of ontogenic changes in GFR may result in potential toxic or subtherapeutic exposureof this drug if the ontogeny of renal function is not accounted for when dosing (Brion et al., 1991).

Dosing must be determined based on the main route of excretion: glomerular filtration or active secretion and if tubular reabsorption plays a significant role. In the first 7 days, serum creatinine may not provide accurate GFR estimates so dosage adjustments can be made based on gestational age (Bartelink et al., 2006; Kramer, 2009). After the first week of life, creatinine levels become a better predictor of GFR through the Schwartz formula (K × L/Scr), which factors in the length (L), serum creatinine (Scr) and a ratio constant (K) that changes based on the child’s age (Bartelink et al., 2006). Using this estimate, one can adjust the dose of drug similarly to the adjustment in renal impairment. If active secretion is significant it can be estimated by comparing total renal clearance values to GFR values and adjustments can be made by the ratio comparison of this value (Kramer, 2009).

## POPULATION PHARMACOKINETICS USING NON-LINEAR MIXED EFFECT MODELS

There are several PBPK modeling programs that are available for use and offer flexibility for pediatric modeling (Björkman, 2005; Bartelink et al., 2006; Läer et al., 2009; Johnson and Rostami-Hodjegan, 2011; Allegaert et al., 2012). One example of the development of PBPK model integrating development changes in ADME that are transformed into one model was proposed by Bartelink et al. (2006). The effects on V_D_, the theoretical volume needed to provide the concentration found in blood plasma for a given dose, largely determine the first dose and effects on clearance determine the maintenance dose. After the dose is adjusted for the effects of V_D_ and clearance initially, further dose modifications need to be made periodically based on changes in the V_D_, clearance and potentially absorption effects. This gives a rational method for dosing and can be built upon with further, top down PBPK methods. PBPK models can also be refined to account for changes in the data that result from development including a developmental shift in the kinetics that alter the rates of elimination, changes in levels of drug metabolites that may cause adverse responses, changes in body composition/weight, and changes in PK that arise from different diseases, e.g., hepatic impairment (Bartelink et al., 2006; Läer et al., 2009; Johnson and Rostami-Hodjegan, 2011; Allegaert et al., 2012). Other factors like changes in GI motility, water intake and output, and food effects can also alter modeling. However, these models does not account for differences in pharmacodynamics, mostly due to fact that very little information exists (Kearns et al., 2003). Ultimately, the PBPK application requires continuous refinement by integrating the results of ongoing research elucidating changes that occur in the neonatal to infant stages of development and *in vitro* information to increase our understanding of dosing schemes (Kearns et al., 2003).

Since Population Based PK approaches are top down methods, they require existing data from a population and utilize data evaluation and iterations on specific covariates to make a dosing decision. To achieve this goal De Cock et al. (2011; Björkman, 2005) have provided a method that uses non-linear mixed effect statistical tools to enable individualized dosing regimens for children of different body weights, ages, and genetic backgrounds. This model is preferred in pediatrics not only because it gives accurate predictions based on a wide variety of covariates, but it also allows for the analysis of sparse datasets that are available as a result of the lack of samples that can be taken in pediatric research. Building these models requires three steps that are listed below (Björkman, 2005; Läer et al., 2009; Johnson and Rostami-Hodjegan, 2011; Allegaert et al., 2012):

(1) Structural model – This decides the specific trend patterns to analyze, how many compartments relevant to the pharmacokinetic study will be used (e.g., central, peripheral, CNS, etc.) and the fixed effects that the model will explore, such as clearance and/or V_D_.
(2) Statistical sub-model – This looks at inter-individual variability between all subjects, and intra-individual variability within individual subjects, along with residual variability.
(3) Covariate sub-model – This step expresses relationships between covariates of which can be individual differences like age, body weight, genetic profile or time-varying differences like renal function, hemodynamic parameters, and body temperature.

From these three models a covariate analysis can be performed to determine what patient specific factors cause a significant difference above the normal variability. Consequently, the optimal dose for a patient can be determined based on the individual characteristics of the patient (Björkman, 2005). It’s important to note that the three models mentioned above are inter-related and the process of finding a model that can adequately describe data is where the challenge lies in these studies. Nonetheless, if this is done properly it can lead to information that demonstrates the impact of developmental changes on pharmacokinetic values.

This model was recently put into action in 2009 by Pfizer’s Global Research and Development team for their patented product Sildenafil, which can be used in children to treat chronic pulmonary hypertension of the newborn and is diagnosed in 1.9 out of 1000 live births (Mukherjee et al., 2009). Sildenafil goes through extensive hepatic metabolism (N-demethylation to UK-103320), primarily by CYP3A4 (79%) and CYP2C9 (20%) and renal clearance is minimal. CYP3A7 may be involved but its role is unclear, therefore rapid development of CYP enzymes may potentially affect sildanefil’s PK during the neonatal period. Clinical trials were done with 36 full-term neonates diagnosed with this condition that were administered intravenous infusions of sildenafil within 72 h at different doses and dose rates (Mukherjee et al., 2009). A mixed effects pharmacokinetic model that included two-compartment disposition of sildenafil and its metabolite’s clearance was used to describe plasma concentration-time data. This model looked at covariates such as postnatal age, weight at screening, and gender compared to the fixed effects of clearance and V_D_. An increase in sildenafil clearance with postnatal age was the only significant covariate effect (Mukherjee et al., 2009). The clearance in the 1-day old subject was 0.84L/h, which based on allometric scaling, is equivalent to 8.05l/h/70 kg or one third of the adult sildenafil clearance (24l/h/ kg). After days 7 the clearance increased to 2.58 L/h, which accurately scales to adult values (Mukherjee et al., 2009). The data was then analyzed by a Top Down population based PK model that incorporated known physiological factors influencing sildenafil using non-linear mixed effects to determine how this parameter is affected based on the covariate of age.

## CURRENT PROGRESS, SHORTCOMINGS, AND FUTURE POTENTIAL

In the last decade, providing children better access to safe and effective medications has become a major agenda of many countries. This has come about due to revolutionary initiatives by WHO, the EU, the WHA, and the FDA that have led to the formation of the international alliance for better medicines for children in 2006 (Gazarian, 2009). The objectives of this alliance are to stimulate research into children’s medicines and to enact systematic strategies to promote quality use of medications (Shirkey, 1968).

In the United States, the legislation to address pediatric needs first came about in 1997 with the FDA modernization act. This ended up becoming a trial and error for the resurgence in pediatric clinical pharmacology and drug development that has occurred from the BPCA and the Pediatric Research Equity Act (PREA) in the past decade (Hoppu et al., 2012). The effectiveness of this legislation has come from providing a mix of requirements and incentives to the pharmaceutical industry. While PREA is required by law, BPCA is voluntary. Under PREA, the FDA and the company determine if a new drug will have indicated use in the pediatric population. If it doesn’t, the requirement is waived and the process ends (Hoppu et al., 2012). If it does have an indication, clinical trials are performed and an agreement is made on the labeling requirements for this product to account for pediatric use. If a drug has PREA tests performed, the company may also have the option to include BPCA tests as well (United States Government Accountability Office, 2011). This voluntary procedure can be done for the new drug or drugs already on the market and is incentivized through a 6 month patent extension on the drug. Through this process, a variety of unlabeled uses can be researched.

A recent analysis of nine antihypertensive medications demonstrated that there was a ratio of net economic return to cost of 17 when these drugs were tested under these provisions (Baker-Smith et al., 2008; Hoppu et al., 2012; Salunke and Breitkreutz, 2013; European Pediatric Formulation Initiative, 2014). Subsequently, this has been very popular and has resulted in “365 trials being performed for 153 medicines between December 1997–2007, which has resulted in 137 labeling changes, 26% of which included the addition of safety information to improve pediatric medicines use” (Hoppu et al., 2012). Despite these successes, the incentives in this legislation are mainly focused on drugs that have patent potential. Many of these drugs have very little use in the pediatric populations, thus many drugs commonly used in children have yet to be sufficiently studied (Hoppu et al., 2012). To stimulate research in this area, the US National Institute of Health, through the Institute of Child Health and Human Development, has refined an approach to facilitate pediatric clinical drug trials through the Pediatric Trial Network that will focus primarily on the study of off-patent medicines prioritized as part of the provisions of BPCA (Hoppu et al., 2012).

Outside of the United States, the impact of this legislation has been minimally successful. Perhaps the greatest triumph has been the establishment of a listing of the most efficacious, safe, and cost-effective medicines for priority conditions, known as the List of Essential Medicines for Children (Hoppu et al., 2012). Beyond that, there has been minimal progress and many countries will not even be able to benefit from the research being performed by organizations from the United States due to the reluctance in sharing data collected on pediatric medicines. To make matters worse, many times the actual research done by US organizations is on foreign soil. So, it appears that volunteers are participating in research that won’t benefit children from their own country. Therefore, despite the successes in producing legislation and results in the USA, these studies indicate many improvements are needed to make this truly a global effort to develop better medicines for children.

While a great deal of research has been done investigating anatomical and metabolism ontogeny and its role in drug ADME, there still remains a large amount of unknowns. Much work has been done in identifying these unknowns by working groups of the Eunice Kennedy Shriver National Institute of Child Health and Human Development (NICHD). Some of the most important knowledge gaps were listed as full understanding of ontogeny of additional drug metabolizing enzymes, transporters, and physiological changes of the GI tract, liver, and kidney (Abdel-Rahman et al., 2012). This illustrates that while much research has been done in this area, our knowledge regarding ontogenic changes is still incomplete. The working group also lists developmental changes in organ perfusion as well as limited literature on aged-based PK of many pharmaceuticals needed to build more accurate PBPK databases.

Clearly, an ontogenic appreciation of organ specific transporter expression and function must be obtained in order to fully understand ADME and Pharmacodynamics across all pediatric age based populations. It would be important to focus efforts on the transporters identified by the International Transporter Consortium (ITC) for their relevance to ADMET first, as a series of decision trees to evaluate function and their role in drug–drug interactions is at the forefront of pharmaceutical research (International Transporter Consortium et al., 2010; Zamek-Gliszczynski et al., 2012, 2013; Giacomini et al., 2013; Tweedie et al., 2013). Several transporter isoforms were selected by the ITC to be of clinical importance for drug development including *P*-glycoprotein (ABCB1), bile salt export protein (ABCB11), multidrug resistance-like protein 2 (ABCC2), breast cancer resistance protein (ABCG2), organic anion transporting polypeptides (SLCO1A2, 1B1, 1B2, and 2B1), organic anion transporters (SLC22A6-8, 11), organic cation transporters (SLC22A1-3), multidrug and toxin extrusion proteins (SLC47A1 and 2; International Transporter Consortium et al., 2010; Zamek-Gliszczynski et al., 2012). It is anticipated that these isoforms would also be of greater priority in pediatric patients.

Of particular relevance to the pharmacogenomic area has been the limited number of case studies where the impacts of genetic polymorphisms in drug metabolizing enzymes and transporters across pediatric patients have been studied. While understanding basal expression levels and function is prerequisite, personalized dosing for pediatric subpopulations is very important to consider for improving therapeutic outcomes. The ITC did investigate some common genetic polymorphisms and concluded that a significant level of evidence supported further investigation into OATP1B1 (c.521T > C, p.V174A, rs4149056) and BCRP (c.421C > A, p.Q141K, rs2231142; Giacomini et al., 2013). Interestingly, a clinical oncology groups study of pediatric patients revealed that OATP1B1 isoforms were present in 1279 out of 1883 cancer patients receiving high dose methotrexate therapy (Ramsey et al., 2013). Lower clearance rates were observed with several polymorphic patients and appeared to be associated with higher toxicity. These outcomes appeared to be most severe with the SLCO1B1 SNP rs4149056 (37041T > C). Dose lowering adjustments and higher hydration rates should be required in children carrying these polymorphic phenotypes. Other studies in children possessing SLCO1B1 polymorphisms were not revealed in our literature search, although their effects on HIV protease inhibitors which are used globally in pediatric patients should be of some concern (Giacomini et al., 2013).

Another NICHD working group has also be established to discuss formulation based changes that must be made for compliant use by pediatric patients (Giacoia et al., 2008, 2012). While this area appears to be outside the scope of this review, it is important to note that formulation changes may very well effect PK and must be taken into account during pediatric drug development. For example, certain excipients can interact with transporters and influence absorption (Constantinides and Wasan, 2007; Werle, 2008; Goole et al., 2010). The European and United States (US) Paediatric Formulation Initiatives (PFIs) have collaborated on establishing the STEP (safety and toxicity of excipients for pediatrics) database to detail risks associated with excipients utilized in formulations for infants, toddlers and children (Salunke et al., 2012, 2013). The effects of these excipients on controlling drug performance needs to be fully assessed with respect to safety and toxicity, which may occur through transporter interactions.

## CONCLUSION

Rational drug dose adjustments to provide children, particularly in the neonatal to toddler age ranges, safe and efficacious exposure must be decided through a comprehensive knowledge of how changes in physiology affect PK during development. Currently our knowledge of many of these factors is limited and a greater effort needs to be made to determine ontogenic factors that influence ADME. Furthermore, development of an understanding of these factors throughout pediatric populations in the world is needed to meet the global challenges facing us. Upon accumulation of this data, the results can be used to refine pediatric PBPK and population based PK models. As these models are further built on more data, dose adjustments may allow targeted clinical research that can be enhanced with the use of non-linear mixed effects models to predict pediatric dosing in specific age-based populations. If pediatric medicines are to reach a level where these populations are no longer considered therapeutic orphans, then the search to find the best treatment options for children must continue through targeted scientific research and the support of legislation to provide incentives to this research. We as a field must take up an advocacy role so that the words of Dr. Shirkey are no longer accurate 50 years from now in 2062, on the 100th anniversary of his infamous assertion.

## Conflict of Interest Statement

The authors declare that the research was conducted in the absence of any commercial or financial relationships that could be construed as a potential conflict of interest.

## Footnotes

1 http://www.translatingtime.net/
